# Supplementary material for: Cognitive Decline Related to Diet Pattern and Nutritional Adequacy in Alzheimer’s Disease Using Surface-Based Morphometry
Source: Nutrients. 2022 Dec 13;14(24):5300. doi: 10.3390/nu14245300 (PMC9784891; doi:10.3390/nu14245300)
Supplement: Supplementary file 1 [file nutrients-14-05300-s001.zip › nutrients-2070536-supplementary.pdf]

Supplementary Data

Table S1. 22 food groups and food items included in this study.

Food group	Food items
Fish	Freshwater fish, marine fish, canned fish
Lean meat	Lean pork, lean beef, lean lamb
Fatty meat	Pork knuckles, pork belly, semi-fatty beef
Poultry	Chicken, duck, goose
Octopus	Shrimp and crab, cuttlefish, octopus, squid, neritic squid
Oyster	Shellfish, snails, oyster
Vegetable	A variety colour of vegetables
Fruit	A variety colour of fruits or fresh juice
Egg	Scrambled eggs, steamed eggs, salted eggs, peeled eggs
Mushroom	Shiitake mushrooms, straw mushrooms, enoki mushrooms, mushrooms
Beans	Red beans, mung beans, vegetable soybean, soybeans and products such as soy milk, bean blossoms, tofu
Soy products	Dried tofu, vegetarian chicken, bean curd strips, fried bean curd
Full-fat milk	Full-fat milk, full-fat goat milk, yogurt
Low-fat milk	Low-fat milk, Low-fat goat milk, yogurt
Skimmed milk	Skimmed milk, skimmed -fat goat milk, yogurt
Fried food	Vegetables, meats, legumes and all other foods that are fried in oil
Processed food	Sausages, jerky, ham, bacon, pork floss
Entrails	The liver, heart, kidneys, large intestine, and small intestine of animals
Sugar	Candy, added sugar such as fructose, sugar, and honey
Sweet drink	Sweetened beverage
Dessert	Snack, dessert, Chinese pasty
Coffee/tea	Coffee, sugar-free green tea, black tea, and oolong tea

Table S2. Cortical thickness data of subjects.

Cortical thickness exam 1(%)	All (n=231)	Female (n=127)	Male (n=109)	p value
Left-Hippocampus[1](%)	0.218±0.041	0.221±0.043	0.216±0.038	0.631
Right-Hippocampus[1](%)	0.225±0.044	0.229±0.046	0.221±0.042	0.192
Left-Amygdala[1](%)	0.078±0.017	0.077±0.018	0.080±0.016	0.190
Right-Amygdala[1](%)	0.096±0.019	0.096±0.020	0.096±0.018	0.818
Left-Accumbens-area[1](%)	0.016±0.007	0.016±0.007	0.017±0.007	0.431
Right-Accumbens-area[1](%)	0.025±0.007	0.025±0.008	0.025±0.007	0.898
**Cortical thickness exam 2(%)**	**All (n=159)**	**Female (n=80)**	**Male (n=79)**	**p value**
Left-Hippocampus[2](%)	0.219±0.038	0.221±0.042	0.217±0.034	0.467
Right-Hippocampus[2](%)	0.225±0.041	0.231±0.043	0.219±0.038	0.072
Left-Amygdala[2](%)	0.079±0.015	0.078±0.017	0.081±0.014	0.328
Right-Amygdala[2](%)	0.096±0.017	0.096±0.018	0.095±0.016	0.907
Left-Accumbens-area[2](%)	0.018±0.007	0.018±0.008	0.018±0.007	0.603
Right-Accumbens-area[2](%)	0.026±0.007	0.027±0.007	0.026±0.006	0.449

[a] Mean ± SD; 1.68E5 represents 1.68×10^5; Mann-Whitney U test for the continuous variables of two groups.

*: p value< .05; **: p value< .01; ***: p value< .001.

Abbreviations: eTIV, estimated total intracranial volume. Measurements with superscript 1 indicate time point correspond to dietary pattern data collection, 2 indicates available historical data with the closest time point with time point 1.

Table S3. Cortical thickness data of the patients by three classified BMI.

Cortical thickness exam1(%)	Underweight [a]	Normal [b]	Overweight [c]	Obese [d]	p value	post hoc
Left-Hippocampus[1](%)	0.199±0.032	0.215±0.037	0.217±0.046	0.226±0.042	0.047	
Right-Hippocampus[1](%)	0.206±0.033	0.220±0.041	0.224±0.049	0.234±0.045	0.086	
Left-Amygdala[1](%)	0.068±0.015	0.075±0.015	0.079±0.020	0.082±0.016	.004**	a<cd; b<d
Right-Amygdala[1](%)	0.088±0.016	0.093±0.020	0.096±0.018	0.100±0.018	0.068	
Left-Accumbens-area[1](%)	0.012±0.005	0.015±0.007	0.017±0.007	0.018±0.007	.009**	a<cd; b<d
Right-Accumbens-area[1](%)	0.021±0.007	0.025±0.008	0.027±0.007	0.026±0.006	.0496*	a<cd
**Cortical thickness exam 2(%)**	**Underweight [a]**	**Normal [b]**	**Overweight [c]**	**Obese [d]**	**p value**	**post hoc**
Left-Hippocampus[2](%)	0.193±0.033	0.218±0.033	0.220±0.039	0.223±0.042	0.261	
Right-Hippocampus[2](%)	0.214±0.030	0.222±0.039	0.224±0.042	0.230±0.043	0.622	
Left-Amygdala[2](%)	0.064±0.016	0.079±0.014	0.081±0.016	0.081±0.015	0.068	
Right-Amygdala[2](%)	0.091±0.012	0.093±0.018	0.097±0.015	0.098±0.017	0.479	
Left-Accumbens-area[2](%)	0.013±0.007	0.017±0.007	0.018±0.007	0.019±0.008	0.098	
Right-Accumbens-area[2](%)	0.023±0.009	0.025±0.008	0.027±0.007	0.027±0.005	0.613	

Mean ± SD; 1.68E5 represents 1.68×10^5; Kruskal-Wallis test for the continuous variables of three groups, Mann-Whitney U test for pairwise comparison; *: p value<.05; **: p value<.01; ***: p value<.001.
Abbreviations: BMI, Body mass index; eTIV, estimated total intracranial volume.
